# From the Pancreas to the Groin: A Scrotal Abscess Following Pancreatic Fully Covered Self-Expandable Metal Stent Placement

**DOI:** 10.7759/cureus.87075

**Published:** 2025-06-30

**Authors:** Jameel Alp, James S Mallery, Guru Trikudanathan, Martin L Freeman

**Affiliations:** 1 Medicine, University of Minnesota, Minneapolis, USA; 2 Medicine/Gastroenterology, University of Minnesota, Minneapolis, USA

**Keywords:** fully covered self-expandable metal stent, inguinal canal abscess, pancreatic duct stenting complication, paracolic gutter abscess, scrotal abscess

## Abstract

Fully covered self-expandable metal stents (FCSEMSs) have gained popularity in the endoscopic management of pancreatic duct strictures, particularly in refractory cases. Although they may offer potential advantages over other stents, recent studies have raised concerns about increased rates of stent-related complications, including migration, secondary strictures, and bile duct obstruction. We report the case of a 49-year-old male with alcohol-induced chronic pancreatitis who developed a rare and extensive infectious complication, including a scrotal abscess, following FCSEMS placement for a benign pancreatic duct stricture. Imaging and culture data suggested a contiguous spread of infection from the peripancreatic region through the paracolic gutter and inguinal canal. This case illustrates a rare but serious complication of FCSEMS use and reinforces the need to avoid their routine use in benign strictures. It also highlights the importance of recognizing underappreciated risks such as side branch obstruction and extrapancreatic infectious spread.

## Introduction

Chronic pancreatitis is a progressive inflammatory disorder characterized by irreversible pancreatic fibrosis, often resulting in chronic abdominal pain, exocrine insufficiency, and endocrine dysfunction. One of its most clinically significant complications is the development of main pancreatic duct strictures, which can lead to obstructive ductal hypertension and persistent pain. While plastic stents remain the standard endoscopic treatment for these strictures, fully covered self-expandable metal stents (FCSEMSs) have emerged as an alternative in refractory cases. However, the use of FCSEMSs carries risks that are not yet fully understood, including rare but severe infectious complications.

## Case presentation

A 49-year-old male with a prior history of alcohol-induced acute and chronic pancreatitis presented to our tertiary care center with worsening epigastric pain and new-onset right groin and scrotal discomfort.

His prior management included multiple endoscopic retrograde cholangiopancreatographies (ERCPs) and stent placements at an outside facility for a benign pancreatic duct stricture. On the initial ERCP, performed approximately a year prior, the stricture was characterized as 30 mm in length and located in the ventral pancreatic duct within the pancreatic head. Upstream of the stricture, the pancreatic duct was diffusely dilated, measuring up to 9 mm in the neck, 6 mm in the body, and 3 mm in the tail, suggestive of functional obstruction.

The stricture had been managed with serial plastic stent placements and gradual upsizing. During the most recent ERCP, again performed at an outside facility, two plastic stents - one 10 Fr × 7 cm and another 7 Fr × 5 cm (Advanix™; Boston Scientific, Marlborough, MA) - were found to be partially occluded. After stent removal, diffuse upstream ductal dilation and irregularity were noted, consistent with marked chronic pancreatitis. This was followed by balloon sweeping, which extracted a few stones, ductal dilation up to 6 mm, and placement of a 10 mm × 6 cm FCSEMS (Viabil®; W.L. Gore & Associates, Flagstaff, AZ) from the ampulla to the pancreatic body. The patient was asymptomatic at the time. Although no rationale was explicitly documented in the procedure note, the choice of stent may have reflected concerns about recurrent occlusion and/or a desire for longer patency. The patient remained asymptomatic and had no episodes of acute pancreatitis following FCSEMS placement until the current admission.

**Figure 1:**
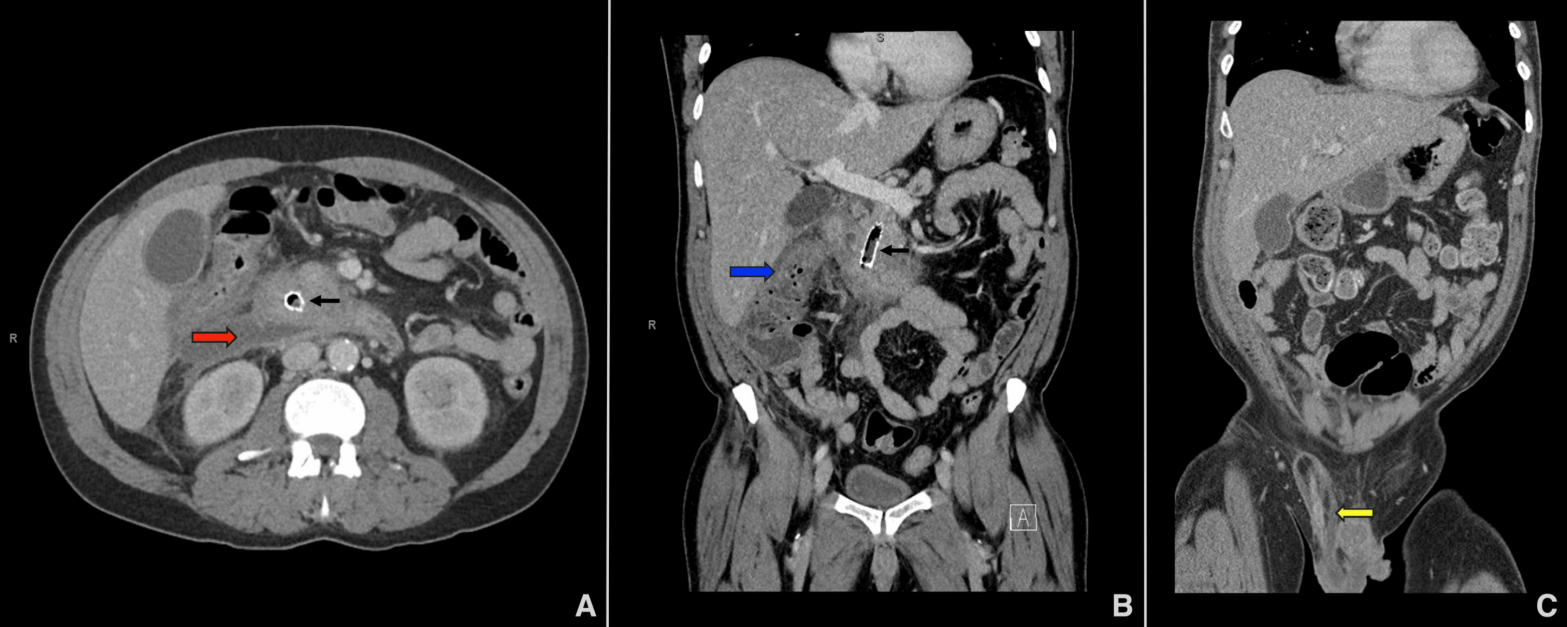
Multiple computed tomography scans revealed extensive abscesses. (A) Infected peripancreatic acute necrotic collection measuring approximately 2.5 × 7.5 × 8.5 cm (red arrow). (B) Contiguous inflammatory changes, including duodenitis and colitis at the hepatic flexure (blue arrow), extending toward the cecum and right hemiscrotum, with a fluid collection measuring approximately 9.8 × 2.9 cm. (C) Abscess formation within the right inguinal canal and spermatic cord (yellow arrow). The fully covered self-expandable metal stent (FCSEMS) is also visible (black arrows).

On presentation, the patient reported consuming 18-24 g of alcohol daily, had a 30-pack-year history of tobacco smoking, and was not on any medications that could cause pancreatitis. He had no history of diabetes mellitus, immunosuppression, prior infections, or evidence of malnutrition. Vital signs were stable. Physical examination revealed a right inguinal hernia without signs of bowel incarceration and epigastric tenderness. Laboratory findings showed a white blood cell count of 23.1 103/µL (normal range: 4.0-11.0 x 103/µL), C-reactive protein of 339 mg/L (normal range: <5 mg/L), and creatinine of 1.54 mg/dL (normal range: 0.6-1.3 mg/dL); liver enzymes and lipase were within normal limits (Table 1). Scrotal ultrasound with Doppler revealed findings suggestive of epididymoorchitis and right inguinal hernia. A computed tomography (CT) scan of the abdomen and pelvis revealed acute-on-chronic pancreatitis with a large peripancreatic fluid collection extending from the head of the pancreas to the hepatic flexure, measuring up to 2.5 × 7.5 × 8.5 cm (Figure 1a). The collection contained internal gas and showed peripheral inflammatory changes, with no evidence of encapsulation. Based on the timing of presentation, absence of a mature wall, and imaging features, the collection was most consistent with an infected acute necrotic collection. The infection extended along the right paracolic gutter into the right lower abdomen and hemiscrotum, forming a contiguous rim-enhancing fluid collection measuring approximately 9.8 × 2.9 cm (Figures 1b-1c). Smaller air- and fluid-containing collections were also seen in the anterior pelvis and along the inferior right rectus musculature. The FCSEMS was in place (Figures 1a-1b), with scattered gas foci within the pancreatic duct and surrounding necrotic collection, indicating infected necrosis confined to the peripancreatic and extrapancreatic regions without necrosis of the pancreatic parenchyma.

**Table 1:** Laboratory values at admission.

Laboratory Test	Patient Value	Reference Range
White Blood Cell	23.1 × 10³/µL	4.0 – 11.0 × 10³/µL
C-Reactive Protein	339 mg/L	<5 mg/L
Creatinine	1.54 mg/dL	0.6 – 1.3 mg/dL

Empiric intravenous antibiotics were initiated with ceftriaxone 2 g daily and oral metronidazole 500 mg three times daily. The patient underwent incision and drainage of the inguinal canal/spermatic cord abscess along with the placement of a percutaneous drain for the necrotic collection and right paracolic gutter abscess. Cultures from all three grew the same organisms, including *Streptococcus anginosus*, *Klebsiella pneumoniae*, and *Fusobacterium nucleatum*, with the exception of *Clostridium perfringens*, which was isolated from the peripancreatic fluid only. Following culture results, the same antibiotic regimen was continued, given adequate coverage and clinical improvement.

**Figure 2:**
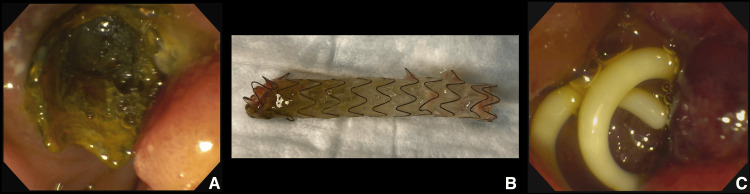
Endoscopic management of the obstructed fully covered self-expandable metal stent (FCSEMS) in the pancreatic duct In the course of the endoscopic retrograde cholangiopancreatography, (A) the FCSEMS (Viabil®; W.L. Gore & Associates, Flagstaff, AZ) was found to be fully obstructed with food, pus, and debris. (B) Following the clearance of all obstructions using a balloon, the FCSEMS was successfully extracted. (C) Subsequently, two short 7 Fr unflanged plastic stents, each with a complete external pigtail (Hobbs Medical, Stafford Springs, CT) were inserted into the main pancreatic duct.

The patient then underwent an ERCP, revealing significant duodenal edema proximal to the major papilla and the FCSEMS obstructed by food particles, purulent material, and debris (Figure 2a). The occluded stent was removed, the pancreatic duct was swept with a balloon, and two short 7 Fr single-pigtail, unflanged plastic stents (Hobbs Medical, Stafford Springs, CT) were placed (Figures 2b-2c).

For the remainder of the hospital stay, the patient underwent multiple interventions, including scrotal exploration, debridement, drainage, orchiopexy, and hernia repair. After source control was achieved via drainage procedures, the patient was discharged on a six-week course of intravenous ceftriaxone and oral metronidazole, to be completed at home via a central line. At his two-month follow-up in the pancreas clinic, the patient reported feeling well and remained asymptomatic. A CT scan showed resolution of all intra-abdominal abscesses, passed stents, and only a mild pancreatic ductal dilation. Given his clinical improvement and absence of symptoms, ERCP was not repeated. He remains under outpatient surveillance with no hospitalizations since discharge.

## Discussion

Endoscopic therapy has become central to the management of pancreatic duct strictures, offering a less invasive alternative to surgery. Plastic stents, particularly 5-10 Fr in diameter with side holes to facilitate drainage of side branches, have long been the standard approach. These are typically placed serially with gradual upsizing, requiring multiple exchanges over a 6-12 month period [1]. However, refractory strictures - often defined as symptomatic strictures persisting beyond one year despite adequate plastic stent therapy - can be difficult to treat [2]. In such cases, FCSEMSs have emerged as a potential alternative due to their larger caliber, increased radial force, and longer patency. Biodegradable stents, which degrade through hydrolysis and potentially reduce the need for removal, are also being explored in Europe but remain unavailable in the United States [3,4].

While several retrospective studies have demonstrated favorable outcomes with FCSEMSs in managing refractory pancreatic duct strictures, including higher stricture resolution and lower recurrence rates, these benefits must be weighed against a higher incidence of adverse events such as stent migration and biliary obstruction [2,5-7]. A noncomparative prospective multicenter trial further highlighted the frequency of adverse events with FCSEMSs [8]. In addition, removal of FCSEMSs can be technically challenging, particularly when the stent lacks a distal retrieval lasso [3]. According to the European Society of Gastrointestinal Endoscopy guideline, temporary placement of FCSEMSs may be considered in refractory strictures [9]; however, this recommendation predates more recent studies reporting increased complication rates. Hence, Strand et al. adopted a more cautious position in the American Gastroenterological Association Clinical Practice Update, noting that, while FCSEMSs may have a role in select cases, further evidence is needed to support their widespread use [10]. Most recently, the American Society for Gastrointestinal Endoscopy recommends against the routine use of FCSEMSs in refractory strictures, citing increased risk of delayed adverse events and uncertain long-term efficacy [1]. These evolving recommendations reflect growing recognition of the risks associated with FCSEMS use.

Prior studies have suggested that the fully covered membrane of metal stents may inadvertently block small ductal side branches, potentially leading to localized ductal hypertension, impaired drainage, and infectious complications [11,12]. In our case, the FCSEMS was found to be completely occluded with refluxed food particles, purulent material, and debris. We hypothesize that, in addition to luminal obstruction, the FCSEMS also contributed to side branch occlusion within the pancreatic duct. Together, these factors likely led to rising intraductal pressure, a side branch leak, and the formation of an extrapancreatic fluid collection. This collection then became secondarily infected, likely through gut bacterial translocation and/or biofilm formation within the stent, and tracked along the right paracolic gutter into the inguinal canal and spermatic cord, ultimately manifesting as a scrotal abscess following gravitational flow.

Several findings support this proposed pathophysiologic sequence. First, CT imaging demonstrated a continuous tract of gas- and fluid-containing collections extending from the peripancreatic region to the inguinal canal and hemiscrotum, consistent with contiguous spread. Second, cultures from the peripancreatic, paracolic, and scrotal collections all yielded overlapping organisms, suggesting a common infectious source. These organisms are part of the gastrointestinal microbiota and are increasingly implicated in pancreatic and peripancreatic infections [13]. Specifically, *Streptococcus anginosus* is associated with abscess formation and biofilm production [14], while *Klebsiella pneumoniae* and *Fusobacterium nucleatum* are known to translocate across disrupted mucosal barriers, contributing to secondary infection [13]. Finally, the patient was asymptomatic prior to FCSEMS placement, and the cascade of complications began shortly thereafter, reinforcing a temporal relationship.

While chronic alcohol use and tobacco smoking may have contributed to baseline pancreatic injury and impaired mucosal defenses, the timing and distribution of the infection point toward a stent-driven process as the dominant mechanism in this case. After removal of the occluded FCSEMS and re-establishment of pancreatic duct drainage with smaller-caliber plastic stents, the patient experienced marked clinical improvement and complete resolution of the necrosis.

## Conclusions

This case highlights the importance of avoiding FCSEMS in non-refractory pancreatic duct strictures and reinforces the use of plastic stents with regular exchanges as the preferred strategy for ductal decompression. Prospective studies are needed to better define the safety profile of FCSEMSs, establish clearer selection criteria, and improve risk stratification for adverse outcomes.
